# Monozygotic multiple pregnancies after transfer of single in vitro produced equine embryos

**DOI:** 10.1111/evj.13146

**Published:** 2019-09-04

**Authors:** A. Dijkstra, J. Cuervo‐Arango, T. A. E. Stout, A. Claes

**Affiliations:** ^1^ Department of Equine Sciences, Faculty of Veterinary Medicine Utrecht University Utrecht The Netherlands

**Keywords:** horse, monozygotic, twins, triplets, in vitro embryo production, intracytoplasmic sperm injection

## Abstract

**Background:**

Monozygotic multiple pregnancy is rare in horses, but may be more common after transfer of an in vitro produced (IVP) embryo.

**Objectives:**

To determine the occurrence, incidence, characteristics and outcome of monozygotic siblings arising from in vivo and IVP equine embryos.

**Study design:**

Retrospective case series.

**Methods:**

A total of 496 fresh in vivo and 410 frozen‐thawed IVP blastocysts, produced by intracytoplasmic sperm injection (ICSI) of in vitro matured oocytes from Warmblood mares, were transferred into recipient mares. The likelihoods of pregnancy and multiple pregnancy were calculated, and the clinical features and outcome of any multiple pregnancy were recorded.

**Results:**

The likelihood of pregnancy after transfer of a single IVP or in vivo embryo was 62% (254/410) and 83% (413/496) respectively. The incidence of multiple pregnancy was 1.6% (4/254) and 0% (0/413) for IVP and in vivo blastocysts, respectively. More specifically, three IVP blastocysts yielded twin embryo propers/fetuses, and one IVP conceptus developed three distinct embryonic bodies. Interestingly, only one embryonic vesicle was detected at all ultrasonographic examinations prior to embryo proper development. Multiple embryonic bodies only became apparent at later scans to check for an embryo proper and heartbeat, or when the recipient mare aborted. Two twin pregnancies aborted spontaneously at 3 and 9 months, respectively, while the heartbeat was lost from all three embryos in the triplet pregnancy before day 35 of gestation. Twin reduction by per rectum compression of one fetal thorax was attempted at day 50 of gestation in the fourth case; however, both fetuses were lost.

**Main limitations:**

Small number of cases.

**Conclusions:**

In vitro embryo production resulted in a higher incidence of multiple monozygotic pregnancy, which could only be diagnosed after development of the embryo proper and is likely to result in pregnancy loss later in gestation if left untreated.

## Introduction

In vitro embryo production (IVEP) by intracytoplasmic sperm injection (ICSI) of in vitro matured oocytes has become an increasingly popular technology in the equine breeding industry because it offers a solution for subfertility of mares and stallions, and allows scarce or expensive frozen semen to be used efficiently 1, 2. The success of IVEP, in terms of ‘number of embryos produced per mare’ often exceeds the availability of recipient mares 3 and this, in combination with out of breeding season production of in vitro produced (IVP) embryos, has made it desirable to cryopreserve these IVP embryos. Moreover, it appears that IVP embryos withstand the freezing process well, presumably because they are yet to expand beyond 250 µm and have yet to form a blastocyst capsule 4, 5. Notwithstanding the potential advantages of IVEP, it is reasonable to suggest that IVEP via ICSI and in vitro culture and/or the cryopreservation process could have some negative consequences. Indeed, there is one report of a single IVP embryo developing into presumed monozygotic twins 6. In this respect, assisted reproductive technologies (ART) in women are known to increase the incidence of monozygotic twins 7, which are often referred to as ‘identical’ because they arise from a single embryo which divides sometime during early development. The incidence of monozygotic twins after transfer of single human IVP embryos has been reported as up to 2.5% 8 compared to 0.4% after natural conception 7. Factors that have been proposed to increase the incidence of monozygotic twinning in human ART programs include the age of the oocyte donor 7, ovarian stimulation, manipulation of the zona pellucida by ICSI or assisted hatching 9, temperature changes (freezing) 7, suboptimal embryo culture media 7, 8, 9 and transfer of a blastocyst versus an early cleavage stage embryo 7. Multiple pregnancies in women are undesirable because they are more likely to be associated with complications such as preterm labour than singleton pregnancies 10. In contrast to ART in women, little is known about the incidence and characteristics of multiple pregnancy after transfer of a single frozen‐thawed IVP horse embryo. This report describes four cases of multiple pregnancy after transfer of a single IVP equine embryo.

## Case descriptions

Cases described in this series were derived from a clinical equine OPU/ICSI program. Briefly, immature oocytes were collected by transvaginal follicle aspiration and shipped overnight at 22°C to an assisted reproductive laboratory where in vitro maturation of oocytes, ICSI and in vitro culture of embryos to the blastocyst stage were performed 11. IVP embryos that reached the blastocyst stage were cryopreserved by slow freezing using 10% glycerol as cryoprotectant 12. To serve as a reference, we also include data from our regular embryo transfer (ET) program in which embryos were flushed 8 days after ovulation and transferred freshly into recipient mares. A total of 410 frozen‐thawed IVP and 496 fresh in vivo embryos were transferred into recipient mares. Fisher’s exact test was used to detect differences in the incidence of monozygotic twin pregnancies between frozen‐thawed IVP and fresh in vivo embryos. The likelihood of pregnancy after transfer of a single IVP or in vivo embryo was 62% (254/410) and 83% (413/496), respectively, and the incidence of detected multiple pregnancy was higher (P = 0.02) for IVP embryos (1.6%, 4/254) than for in vivo embryos (0%, 0/413). More specifically, three IVP embryos developed into a twin pregnancy after transfer, while one IVP embryo developed into a triplet pregnancy. A more detailed description of the four cases can be found below.

### Case 1

**Figure 1:**
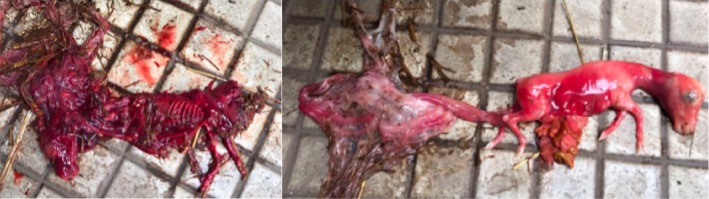
Mummified (left) and freshly aborted fetus (right) at 95 days of gestation, and their remaining fetal membranes.

A multiparous Warmblood recipient mare with a foal‐at‐foot was transported to Utrecht University 5 days after ovulation for transfer of a frozen‐thawed IVP embryo from a 23‐year‐old Warmblood donor mare. A single embryonic vesicle was detected by transrectal ultrasonography 10 days after ET, equivalent to approximately day 15 of gestation. The mare was next examined by a referring veterinarian at 88 days of gestation; this confirmed that the mare was still pregnant, although the existence of twins was not established. The mare aborted two fetuses one week later; one of the aborted fetuses was mummified (Fig 1).

### Case 2

**Figure 2:**
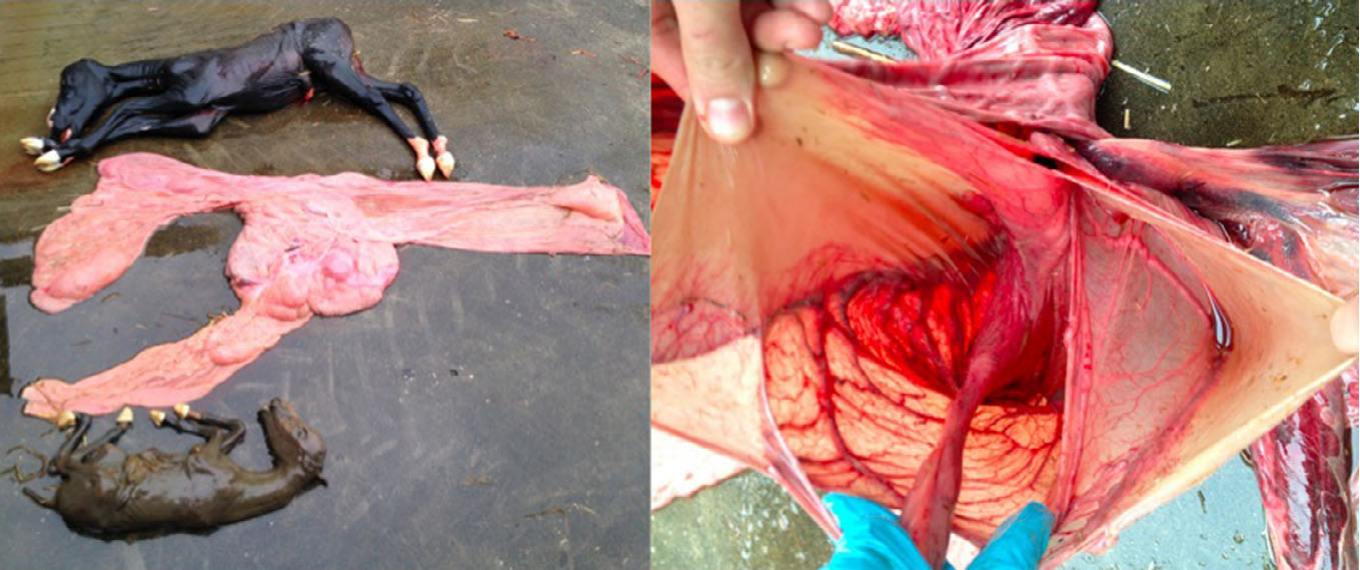
Gross appearance of the two fetuses and placenta aborted at 267 days of gestation. One fetus (top) had a fresh appearance while the smaller fetus (bottom) was in the process of mummification. The appearance of the fetal membranes was unusual, with the individual allantoic sacs apparently bounded by a single shared chorion indicating a monochorionic, diallantoic twin pregnancy. The origins of the two umbilical cords were adjacent.

A cryopreserved IVP blastocyst from a 24‐year‐old Warmblood donor mare was thawed and transferred to an 8‐year‐old barren Warmblood recipient mare 5 days after ovulation. Immediately after ET, the recipient mare was picked up by the owner and all pregnancy examinations were performed by a referring veterinarian. The first pregnancy examination was performed 10 days after ET and revealed a single embryonic vesicle. At day 45 of gestation, a single viable fetus was detected after which the examination was discontinued because the veterinarian did not expect twins after ET. At 267 days of gestation, the recipient mare aborted twins; the placenta and fetuses were taken to Utrecht University for examination, which revealed a monochorionic, diallantoic twin pregnancy (Fig 2).

### Case 3

**Figure 3:**
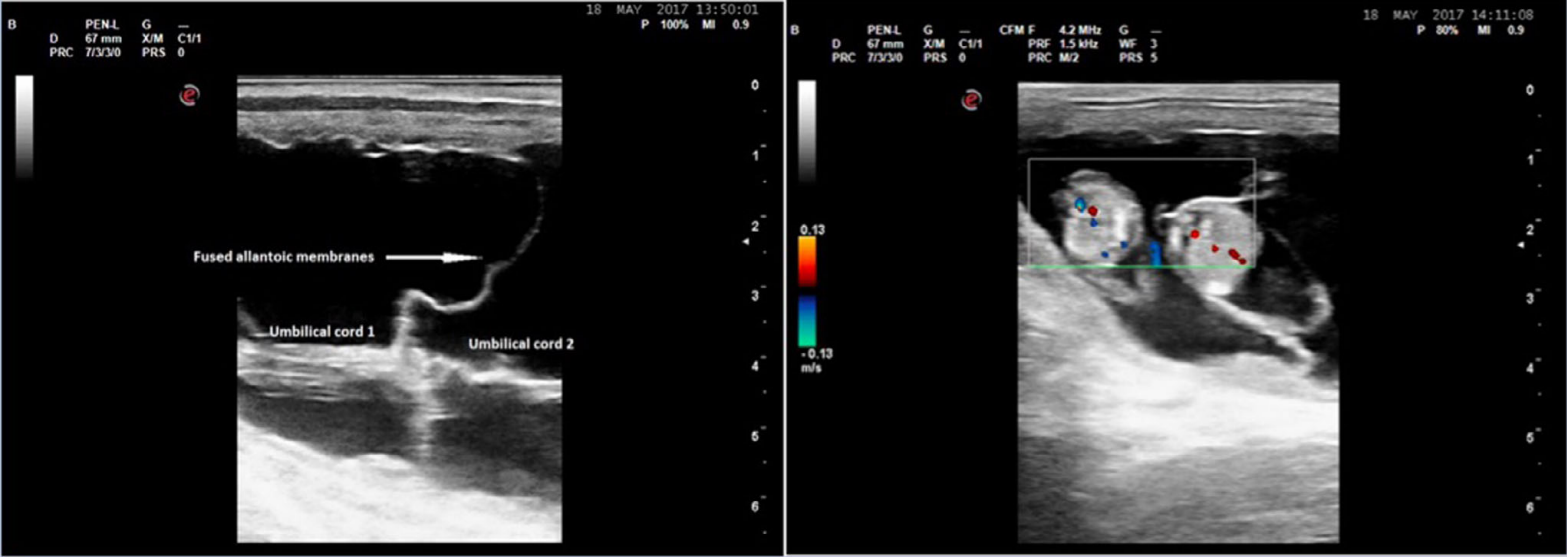
Ultrasonographic image of the twin pregnancy at 50 days of gestation. The umbilical cords of the two fetuses appear to fuse at the point at which the allantoic membranes abutted (left image). Both fetuses are similar in size (right image).

A cryopreserved IVP embryo from a 17‐year‐old Warmblood donor mare was thawed and transferred into a recipient mare on day 5 after ovulation. Pregnancy was confirmed 10 days after ET by transrectal ultrasonography during which a single embryonic vesicle was detected. At 33 days of gestation, the pregnancy was rechecked and found to contain two embryonic bodies each with their own allantois but sharing the yolk sac, indicating a monochorionic, diallantoic pregnancy. The recipient mare was examined again at day 46 of gestation when two viable fetuses were detected. The recipient mare was presented to Utrecht University for attempted twin reduction 4 days later. Each fetus had its own allantois and umbilical cord. However, the umbilical cords of the two fetuses appeared to fuse at the point at which the allantoic membranes abutted (Fig 3). The two fetuses were similar in size (Fig 3) and the thorax of one fetus was manually compressed between the ultrasound probe and the ventral pelvis until its heart stopped beating. Altrenogest (0.088 mg/kg bodyweight) was then administered once daily for 7 days. The recipient mare was re‐examined 1 week after attempted twin reduction but had lost both pregnancies.

### Case 4

**Figure 4:**
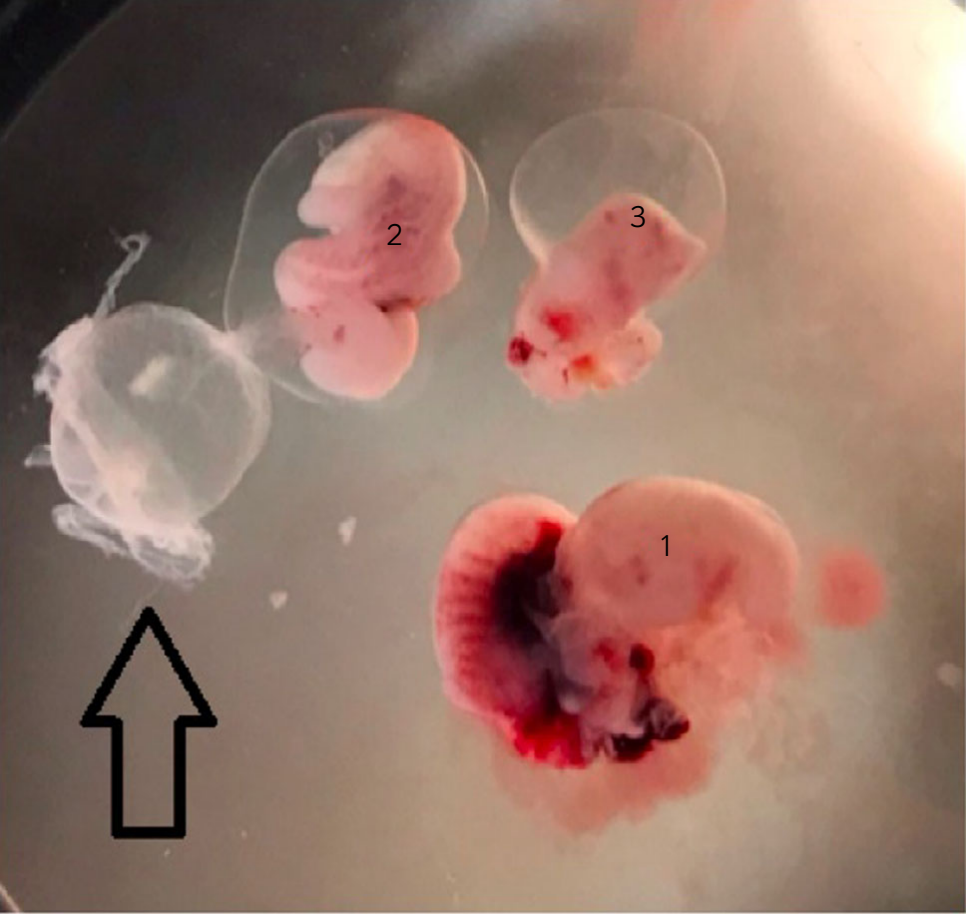
Gross appearance of the three embryos from a dead day 35 conceptus, using a dissecting microscope. The largest embryo (nr 1) had some areas of haemorrhage and had ruptured in the mid‐body region. The second embryo (nr 2) was intermediate in size, surrounded by an amnion and was dissected with its allantois still attached (arrow). The smallest embryo (nr 3) had small focal haemorrhagic lesions and was also partially contained within its amnion. All three embryos had a visible head and eyes.

A cryopreserved IVP blastocyst from a 10‐year‐old competing Warmblood donor mare was thawed and transferred into a 7‐year‐old recipient mare 4 days after ovulation. Pregnancy detection was performed 1 week later, but no embryonic vesicle was detected. Two days later, a single 3.9 mm embryonic vesicle was detected in the uterine body. The recipient mare was examined again 1 week later (day 16 after ET) when a single 29 mm embryonic vesicle was detected at the base of the right uterine horn, although no embryo proper was detectable. Seven days later (23 days after ET), two embryo propers both with a heartbeat were detected; however, the volume of yolk sac fluid was less than expected for a normal pregnancy. Two days later, the heartbeat of both embryos was slow, and by day 30 after ET no heartbeat was detectable in either embryo. The conceptus was flushed out of the uterus using lactated Ringer’s solution and a sterile endotracheal tube with an internal diameter of 20 mm. The vesicle was evaluated using a dissecting microscope which revealed the presence of a single yolk sac but three separate embryonic bodies, with distinct head and eyes, and each of which had its own amniotic sac and developing allantois (Fig 4).

None of the recovered conceptuses or aborted fetuses in any of the cases were genotyped to confirm that they were genetically identical. Nonetheless, each IVP embryo was produced by injecting a single spermatozoon into an oocyte. Furthermore, none of the recipient mares were exposed to a stallion prior to ET.

## Discussion

Roberts *et al*. 6 described series of ultrasound examinations of a twin pregnancy that developed after ICSI that they presumed to be monozygotic. This report expands upon the possibility of monozygotic multiple pregnancy after IVEP by describing the incidence and characteristics of monozygotic twins and triplets after transfer of single frozen‐thawed IVP equine embryos. The likelihood of multiple pregnancy was higher after transfer of a single frozen‐thawed IVP embryo (1.6%) than after transfer of a single in vivo embryo (0%). Although we did not record any multiple pregnancies after conventional ET, it has been reported to occur after transfer of in vivo developed equine embryos recovered by traditional embryo flushing 13, 14. McCue *et al*. 13 reported three cases of multiple pregnancy after transfer of 767 in vivo embryos. Based on our data, it is impossible to determine whether this apparently increased incidence of multiple pregnancy in mares is real and can be attributed to either IVEP or the cryopreservation process. Nevertheless, data from human IVF programs suggest that it is more likely to be linked to IVEP than to cryopreservation 7. Indeed, a similar increase in the incidence of monozygotic twins has been observed after transfer of fresh IVP human embryos 8, and manipulation of the zona pellucida by ICSI and suboptimal culture conditions were proposed as predisposing factors for the development of monozygotic twins 7.

The fact that a single IVP embryo can give rise to a multiple pregnancy is intriguing and, therefore, several hypotheses have been proposed to explain the underlying mechanism 7. One hypothesis is that part of the inner cell mass (ICM) adheres to the opposing inner wall of the blastocyst during embryo collapse, resulting in division into two ICMs after re‐expansion. In the present case series, embryo collapse could have occurred during the cryopreservation process. On the other hand, the degree of embryo collapse is limited in IVP equine blastocysts because they are small (<200 µm) and contain a poorly discernible blastocoel. Moreover, collapsing large (300–1000 µm) in vivo equine blastocysts by aspirating blastocoel fluid prior to cryopreservation does not appear to result in an increased incidence of multiple pregnancy 15. Another hypothesis is that part of the ICM and trophectoderm herniate/hatch through the opening in the zona pellucida created by the Piezo drill during ICSI. The hatched part of the IVP embryo can either remain connected to the rest of the embryo or become detached from the original embryo after transfer into the uterus of a recipient mare. If the hatched part becomes dislodged and contains sufficient cells (including ICM cells), two physically separate embryos would develop within the uterus. However, in the current case series, only one embryonic vesicle was detected at the first pregnancy examination, making this explanation unlikely. The third hypothesis is that either ICSI or/and in vitro culture (media and conditions) has a direct effect on the developing embryo causing the development of two ICMs within the zona pellucida 7, or at a later stage the epiblast splits to form two embryonic discs 16. Consequently, two separate embryonic discs develop within the same vesicle, resulting in a single embryonic vesicle with two embryo propers, each of which develops its own amnion and allantois. The final possibility is that a single embryonic disc forms, but that two primitive steaks arise within the same disc; this would be expected to result in monochorionic, monoamniotic pregnancies 17. Regardless of the underlying mechanism, these ‘twin’ IVP embryos cannot be identified at the time of ET because the ICM of an IVP equine embryo cannot easily be distinguished from the trophectoderm cells using a dissecting microscope.

This case series emphasises the importance of pregnancy examination after expected development of the embryo proper, even following transfer of a single embryo, because only one embryonic vesicle was detected by ultrasonography 7–10 days after transfer of an IVP equine embryo in at least two of the cases. Although a single embryonic vesicle was also reported in the other two cases, it is not possible to rule out the presence of a second embryonic vesicle during the mobility phase, not least because none of the follow‐up pregnancy examinations revealed a twin either. In this respect, it appears that the twins were missed by the referring veterinarians at 45 and 88 days of gestation, respectively, primarily because the existence of twins was not considered possible. The presence of a single embryonic vesicle before appearance of the embryo proper has also been described in the majority of the multiple pregnancy cases after transfer of a single in vivo equine embryo 13, 14 indicating that it is more the rule than the exception.

The outcome of a multiple pregnancy derived from a single IVP embryo was poor; both mares with undiagnosed twins aborted while the triplet pregnancy died spontaneously during the embryonic period. However, loss of the triplet pregnancy could also be a result of poor quality of the IVP embryo rather than a direct consequence of the multiple pregnancy since the embryonic vesicle in this case was ‘small for age’ having been first detected as an unusually small vesicle on day 9 after ET. We recently reported that a small embryonic vesicle on day 7 after transfer of an IVP embryo was associated with an increased incidence of early embryonic loss 12. Similar to our findings, all cases of multiple pregnancy after transfer of single IVP (n = 1) and in vivo (n = 6) equine embryo in other reports resulted in either early pregnancy loss or late‐term abortion 6, 13, 14. Since twin reduction was only attempted in one set of twins, we can only speculate on the likely success of twin reduction. There are several reasons to suggest that the outcome of twin reduction is likely to be poor in monochorionic twins. Firstly, the presence of a single embryonic vesicle prior to appearance of the embryo proper excludes the possibility of twin reduction prior to vesicle fixation. Furthermore, even though two embryonic bodies develop, only one yolk sac will be present making reduction of one embryo/fetus between days 25 and 35 without damaging the second embryo extremely challenging. Finally, if all of these multiple pregnancies really are monochorionic but diallantoic they must share a chorion, and it is unclear to what degree demise of one fetus would affect the viability of its co‐twin, which by analogy to human monochorionic twin pregnancies would be expected to have joined blood vessels and therefore communicating placental circulations. Although there is a report of a live foal born after injecting potassium chloride into the heart of its monozygotic twin, this twin pregnancy was derived after transfer of a single in vivo equine embryo but was classified as dichorionic 18.

In conclusion, in vitro production of horse embryos by ICSI and in vitro culture appears to be associated with an increased incidence of monozygotic twinning. These monozygotic twins appear to be monochorionic and can, therefore, only be diagnosed after development of the embryo proper. Although the exact underlying mechanism is unknown, it is likely the ICM divides within the early embryo or generates more than one primitive streak. Irrespective of the mechanism, the outcome of those multiple pregnancies is poor.

## Authors’ declaration of interests

The authors have no competing interest.

## Ethical animal research

Research ethics committee oversight not required by this journal: retrospective case series.

## Owner informed consent

Owners of mares included in the case series gave consent for their animals' inclusion.

## Authorship

A. Dijkstra and A. Claes contributed to study design. A. Dijkstra, A. Claes, J. Cuervo‐Arango and T. Stout contributed to study execution, data analysis and interpretation, and preparation of the manuscript. All authors gave their final approval of the manuscript.
